# Nongenetic Individuality in the Host–Phage Interaction

**DOI:** 10.1371/journal.pbio.0060120

**Published:** 2008-05-20

**Authors:** Sivan Pearl, Chana Gabay, Roy Kishony, Amos Oppenheim, Nathalie Q Balaban

**Affiliations:** 1 Racah Institute of Physics, Hebrew University, Jerusalem, Israel; 2 The Center for Nanoscience and Nanotechnology, Hebrew University, Jerusalem, Israel; 3 Department of Molecular Genetics and Biotechnology, Hebrew University-Hadassah Medical School, Jerusalem, Israel; 4 Department of Systems Biology, Harvard Medical School, Boston, Massachusetts, United States of America; Harvard University, United States of America

## Abstract

Isogenic bacteria can exhibit a range of phenotypes, even in homogeneous environmental conditions. Such nongenetic individuality has been observed in a wide range of biological processes, including differentiation and stress response. A striking example is the heterogeneous response of bacteria to antibiotics, whereby a small fraction of drug-sensitive bacteria can persist under extensive antibiotic treatments. We have previously shown that persistent bacteria enter a phenotypic state, identified by slow growth or dormancy, which protects them from the lethal action of antibiotics. Here, we studied the effect of persistence on the interaction between Escherichia coli and phage lambda. We used long-term time-lapse microscopy to follow the expression of green fluorescent protein (GFP) under the phage lytic promoter, as well as cellular fate, in single infected bacteria. Intriguingly, we found that, whereas persistent bacteria are protected from prophage induction, they are not protected from lytic infection. Quantitative analysis of gene expression reveals that the expression of lytic genes is suppressed in persistent bacteria. However, when persistent bacteria switch to normal growth, the infecting phage resumes the process of gene expression, ultimately causing cell lysis. Using mathematical models for these two host–phage interactions, we found that the bacteria's nongenetic individuality can significantly affect the population dynamics, and might be relevant for understanding the coevolution of bacterial hosts and phages.

## Author Summary

Persistence of subpopulations of bacteria to antibiotic treatments is a major problem in recurrent infections. Unlike resistance, which is passed on to the next generations, persistence is a transient trait characterized by slow growth or dormancy. It has been suggested that the existence of both persister and non-persister bacteria within a given population might constitute a general strategy that bacterial populations use to cope with an ever-changing, stressful environment. Here, we studied the influence of persistence on the interaction between bacterial populations and viruses that infect bacteria, called phages. We found that persistence provides a clear advantage for lysogenic bacteria—in which the phage DNA has integrated into the host DNA but remains mostly inactive—as they enter the reversal of this state, typically in response to environmental stress. This suggests that persistence might have evolved in lysogenic bacteria under stressful conditions. In contrast, persister bacteria do not survive infections by lytic phages—which replicate until they cause the host cell to burst—any better than non-persister bacteria, but release the infectious phages on a significantly longer time scale. Mathematical analysis reveals that this host heterogeneity might substantially affect host–phage population dynamics and could be relevant for other predator–prey systems.

## Abstract 3

Mathematical analysis and single-cell observations of bacteria persistent to antibiotic treatments shed new light on the ecology of bacteria and phages.

## Introduction

**Figure 1:**
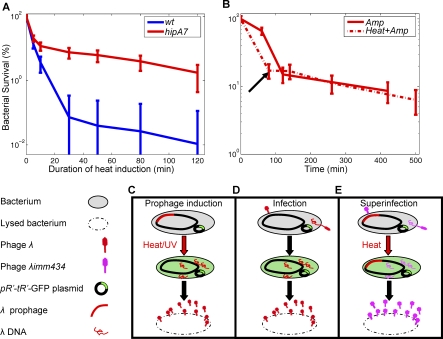
High Persistence in the Response to Prophage Heat Induction (A) Cultures of wt and *hipA7 λcI857KnR* lysogenic bacteria were subjected to heat (42 °C) to trigger prophage induction. The survival percentages are plotted versus the duration of exposure to heat. The killing curves show the bimodal behavior characteristic of persistence, with the *hipA7* persistence level far above the wt. (B) Persisters to prophage heat induction persist ampicillin (Amp) treatment: Killing curves in ampicillin of *hipA7 λcI857KnR* lysogenic bacteria (solid line) and of persisters to 80-min prophage heat induction (dashed line). Arrow shows the time of transition from heat induction to ampicillin exposure. The two curves are similar, showing that persisters to prophage heat induction are also persistent to ampicillin. Error bars represent the standard deviation between triplicates. Similar results were obtained in at least three independent experiments. (C–E) Schematic layout of host–phage systems used in this work (C) Prophage induction of *λcI857KnR* lysogens by heat, or of *λc+KnR* by UV. (D) Infection by *λcI60.* (E) Heat-induced *λcI857KnR* lysogens superinfected with *λimm434cI3003* phages.

Fifty years ago, studies on the heterogeneity of genetically uniform populations demonstrated the importance of single-cell individuality for understanding a number of phenomena, including enzyme induction and radiation resistance [1–3]. The importance of heterogeneity is evident in the response of bacterial populations to antibiotic treatments, in which most bacteria are rapidly killed, but small subpopulations nevertheless persist [4]. Recently, a renewed interest in the persistence phenomenon has revealed that nongenetic heterogeneity might be one of the main reasons for the failure of antibiotic treatment in infections such as tuberculosis, in which a single persistent bacterium can restart an infection [5,6]. Persistence is typically observed through the monitoring of the survival fraction of a bacterial population exposed to antibiotics. A curve showing an initially rapid killing of the bacteria, followed by a significantly reduced killing rate indicates the presence of a persistent subpopulation (Figure 1B, solid curve). When cells grown from this persistent subpopulation are subjected again to antibiotics, the same biphasic killing curve is obtained, suggesting that the persistent subpopulation is not genetically different from the original population. Several mutants with high persistence (*hip*) to a range of antibiotics were isolated [7,8]. Previously, we directly observed single persister cells in *hip* strains and determined that persistence is due to an inherent heterogeneity of growth rates in the E. coli population that existed before the antibiotic treatment [9]. Two different processes generate persister cells in the population. In Type I persistence, persister cells are generated when the culture reaches stationary phase [10]. Once transferred to fresh medium, the inoculum contains both normal and persister cells. While the normal cells present in the inoculum start growing within half an hour, persister cells remain dormant for periods that may exceed a day. Because Type I persisters appear at stationary phase and not during the subsequent exponential growth, their number depends on the size of the inoculum from stationary phase [9,11]. Type I persisters exit their dormant state stochastically and switch to normal growth [9]. In contrast, Type II persisters are continuously generated during exponential growth and do not require a starvation signal. The equations describing the dynamics of switching between the normal and persister states have been described for both persistence types [9]. In our present work, we focus on Type I persistence, which has been identified as a major factor of persistence to antibiotics in wild-type (wt) E. coli, as well as in Staphylococcus aureus and in Pseudomonas aeruginosa [11].

In view of the fact that phenotypic individuality plays an important role in bacterial persistence to antibiotics, we were curious to examine its possible involvement in the context of the interaction between bacteria and phages. The abundance of phages in various ecological niches [12] suggests that they represent one of the most common stresses that bacteria have encountered during evolution. Recent studies have indeed revealed the important role played by phages in the evolution [13] and ecology of bacteria [13]. For example, phages are believed to play a pivotal role in the control of bacterial populations in oceans [14]. Our goal was to study the effect of the dormant subpopulation of Type I persisters on the bacterial–phage interaction. We wished to determine whether bacteria persistent to antibiotic treatment are also protected from phage-mediated lysis. For this purpose, we compared the wt *E. coli* MG1655 to its *hipA7* derivative. The *hipA7* mutation was shown to confer a high level of Type I persistence [15]. It consists of two point mutations located in the *hipA* gene of the *hipBA* operon [10], which acts as a toxin–antitoxin module [16,17]. We adapted our methodology, originally developed for the study of persistence to antibiotics, to investigate the nongenetic individuality in the interaction between E. coli and the λ-phage The λ-phage is a temperate phage that infects E. coli by injecting its DNA into the bacterium. Upon infection, it chooses between lysogeny, in which the phage embeds its genome into the bacterial chromosome and is carried on as prophage in the division process, or lysis, in which the phage activates genes that enable its replication and lysis of the host [18]. Activation of the lytic pathway in lysogenic bacteria, namely prophage induction, can be triggered by stressful conditions such as DNA damage.

Using long-term single-cell observations, we studied the ability of persister bacteria to survive phage assaults. We investigated both the induction of prophages and lytic infections.

## Results and Discussion

### Persister Bacteria Survive Prophage Induction

We first compared prophage induction in wt and in the *hipA7* mutants with increased persistence to antibiotic treatment. Prophage induction was studied in populations of lysogenic bacteria carrying prophage *λcI857KnR.* The *λcI857KnR* phage carries temperature-sensitive mutations in CI, the lytic pathway repressor, that lead to the inactivation of repression at nonpermissive temperatures (42 °C) and to the initiation of the lytic pathway [19]. This system makes possible the controlled induction of prophages (Figure 1C). We subjected MG1655 (wt) *λcI857KnR* and MG1655A7 (*hipA7*) *λcI857KnR* lysogenic strains to prophage induction at 42 °C. The fraction of survivors versus the duration of heat induction is shown in Figure 1A. Similar to the persistence to antibiotic treatments, the survival under prophage induction was much higher for the *hipA7* strain than for the wt. A substantial percentage of the *hipA7* lysogenic bacteria persisted, even after several hours at 42 °C. When the progeny of these persisters was again exposed to 42 °C, similar killing and survival rates were observed, indicating that persisters to phage induction did not originate from resistant mutants (Text S1).

**Figure 2:**
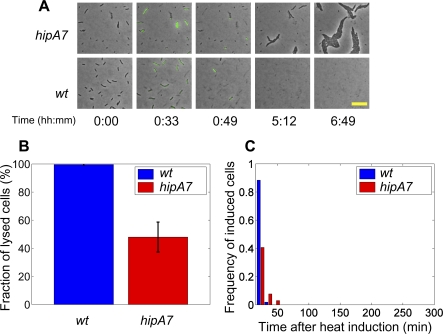
Single-Cell Observation of Persistence Following Heat Induction for Wild-Type and *hipA7 λcI857KnR* Lysogens (A) Superposition of phase-contrast and fluorescence images acquired by time-lapse microscopy of wt and *hipA7* strains carrying *λcI857KnR* prophage during and after heat induction. At *t* = 0, lysogenic bacteria are exposed to 42 °C for 80 min. The temperature is then set back to 32 °C. Videos were acquired in parallel at several different locations. At the end of the measurements, no survivors are observed in the wt, whereas a number of dividing cells can be seen in the *hipA7* strain. Scale bar indicates 20 μm. (B) Percentage of lysis following heat induction, based on phase microscopy over the total observation period (8 h). More than 50% of the *hipA7* population does not lyse, showing the existence of persisters. Error bars represent the standard deviation between two separate microscopy experiments, on a total of approximately 1,400 cells for each strain. (C) Monitoring of late promoter *pR′-tR′-gfp* onset of fluorescence (in lysing cells only). Histograms are normalized to the total number of cells that were followed individually (approx. 250 cells each). Onset of fluorescence occurs within 1 h following heat induction, and no induction events were observed in the subsequent 4 h of continuous observation.

These results were substantiated by single-cell observations: Figure 2A shows the time course of a typical experiment, in which we exposed *hipA7* and wt lysogenic bacteria to 42 °C under the microscope, and followed their response, simultaneously. The activation of the lytic pathway was monitored by measuring the fluorescence level of green fluorescent protein (GFP) expression under the control of *pR′-tR′*, the promoter of the late lytic genes [20]. Whereas the wt population was massively induced and lysed within the first hour following heat induction (Video S1), only 40% of the *hipA7* population (Video S2) was induced and lysed during the total observation time (Figure 2B). After 80 min at 42 °C, the temperature was shifted back to 32 °C. Bacteria that survived the heat induction did not express the lytic pathway genes, and started dividing, forming microcolonies (Figure 2A). We observed, under the microscope, that those cells were neither growing nor dividing during the heat-induction treatment. Bacteria that did respond to phage induction in the *hipA7* strain followed the same dynamics of induction as wt cells, as seen by monitoring the increase in fluorescence expression from the *pR′-tR′-gfp* fusion (Figure 2C). Thus, the response to prophage induction of *hipA7* lysogenic bacteria reveals two subpopulations: persister bacteria, which are not induced and survive prophage heat induction, and normally growing bacteria, which follow the same induction dynamics as the wt cells and lyse. As expected from Type I persistence, survival following prophage induction decreased with the inoculum's size, falling below 0.1% for an inoculum of 10^3^ bacteria (Figure S1). Furthermore, when we exposed the survivors following prophage induction to ampicillin treatment, we observed the expected low killing rate (Figure 1B), indicating that persisters to prophage induction are also persistent to ampicillin.

High persistence was also observed for the induction of the wt λ-phage by UV irradiation (Figure S2), when UV-irradiated lysogenic bacteria were subsequently grown under illumination to allow for photoreactivation [21] (Text S1). Collectively, these results show the correspondence between persistence to antibiotics and to prophage induction, and suggest that dormancy protects persisters from both stresses. This view is supported by previous studies on the influence of the host physiology on prophage induction [22].

### Absence of Persistence under Lytic Infection

We now turn to the response of the *hipA7* strain to infection by the λ-phage. Are persisters as protected from phage infection as they are from prophage induction? To answer this question without involving the additional complication of the lysis–lysogeny switch, we subjected *hipA7* and wt strains to *λcI60* infection (Figure 1D). The *cI60* mutation allows infection and lysis, but prevents the formation of lysogens by disabling CI repression. Survival to *λcI60* infection was measured by counting colony forming units (CFU) on plates, as well as by observation under the microscope. In contrast to the higher survival of *hipA7* bacteria to prophage induction, after infection, we measured low bacterial survival in both the wt and *hipA7* strains, to the rate of 0.09% ± 0.11% and 0.04% ± 0.03%, respectively. Under the microscope, we observed lysis in more than 90% of the cells, for both strains, over a total observation time of several hours. We found that the observed killing was due to the initial exposure to the phage during adsorption and not to a secondary infection process after plating (see Text S1). Thus, *λcI60* phages are able to infect and kill persister bacteria, despite their dormancy.

### Killing Occurs upon Switching from Persistent to Normal State

**Figure 3:**
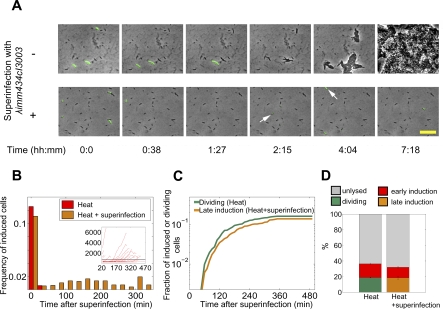
Exit from Dormancy Is Required for Phage-Mediated Lysis (A) Superposition of phase-contrast and fluorescence images acquired by time-lapse microscopy of the *hipA7 λcI857KnR* lysogens after heat induction, without (upper panel) and with (lower panel) *λimm434cI3003* phage superinfection. Fluorescence expression of the *pR′-tR′*-*gfp* fusion is monitored. At the end of the measurements, no survival is observed in the superinfected cells (namely, no dividing cells), whereas several dividing cells can be seen in the noninfected cells. The arrows point to the location of a superinfected bacterium with late onset of fluorescence. Scale bar indicates 20 μm. (B–D) Single-cell analysis of *hipA7 λcI857KnR* lysogens following heat induction, with and without phage superinfection. (B) Timing of the onset of *pR′-tR′*-*gfp* activation. Histograms are normalized to the total number of cells (approx. 1,000 cells each). The initial fast response is analogous to the one presented in Figure 2C and is followed here by a clear spread of onset activation times in the superinfected cells. Inset: typical single-cell fluorescence expression curves. Onset time is defined as where the fluorescence crosses the threshold (black line). (C) Exit from dormancy of superinfected and noninfected lysogens. Persisters to heat induction in both conditions were followed for either onset of fluorescence (superinfected), or division (noninfected). The time dependence of both processes is similar, showing that exit from dormancy is required for activation of the lytic program. (D) Relative percentage of the different outcomes of heat induction, with and without phage superinfection, observed by phase and fluorescence microscopy. Error bars represent the standard deviation between two separate microscopy experiments.

How do phages kill persister bacteria? Are they able to activate the lytic pathway during the persister's dormancy, or do they initiate the lytic pathway only upon switching from dormant to growing cells? If the latter is true, the onset of the lytic pathway activation in persister bacteria should follow the same time dependence as the exit from dormancy. We followed the dynamics of lytic gene expression in single infected persister cells by tracking the GFP fluorescence expression from the λ late promoter *pR′-tR′* fused to *gfp*. In addition, we simultaneously measured the dynamics of persisters' exit from dormancy. Initially, we identified persisters as follows: we subjected *hipA7 λcI857KnR* lysogenic bacteria to 30-min heat induction at 42 °C, as described above, in order to rapidly induce and kill normally growing bacteria. We then measured the exit of persisters from dormancy by identifying their first division with phase-contrast microscopy. Simultaneously, we measured the onset of fluorescence due to lytic pathway activation in persisters infected with lytic phages (Figure 1E). To overcome the immunity of *λcI857KnR* to superinfection, we used a different immunity phage, *λimm434cI3003*, that can superinfect *λcI857KnR* lysogenic bacteria and, similar to *λcI60*, does not form lysogens [23]. Figure 3A shows the time course of a typical experiment, in which we observed *hipA7 λcI857KnR* lysogens after 30-min exposure to 42 °C without infectious phages (upper panel), and with *λimm434cI3003* superinfection (lower panel). Without superinfection, persister cells exited dormancy and formed microcolonies, as shown above (Figures 2A and 3A, upper panel); with superinfection, no survival was detected, but we observed a late onset of fluorescence in persister bacteria (Figure 3A, arrows in lower panel), indicating that the phage lytic cycle was activated. Using automated image analysis, we quantified the timing of onset of fluorescence activation. The histograms for superinfected and noninfected lysogens are presented in Figure 3B. A rapid activation of fluorescence was observed in both conditions, identifying normally growing bacteria. However, after about 1 h, persister cells that were superinfected began increasing their GFP expression from the *pR′-tR′* promoter and lysed. These late-activation events of the lytic promoter lasted until the end of our experiments (Video S3). In Figure 3C, we now compare the late onset of fluorescence, in superinfected cells, to the timing of exit from dormancy, in persisters without superinfection. The correspondence of the timing of these events is striking. Furthermore, the percentage of single cells showing late GFP fluorescence activation was similar to that of noninfected persisters that exited dormancy and divided, within the same observation time (Figure 3D).

These results show that only when infected persisters exit dormancy, activation of the phage genes starts and lysis follows. This suggests that, despite the large physiological differences between normally growing and stationary bacteria [24,25], phages are able to infect and eventually to lyse their hosts in both physiological states, showing the perfect adaptation of phages to their host. This behavior is reminiscent of the strategy observed for several phages that can infect starved bacteria, or in phages of sporulating bacteria. For example, under nutrients limitation, T4 phages can enter a state of “hibernation” and activate the phage program only once nutrients become available. Similar to pseudolysogeny, these phenomena might provide an explanation for the maintenance of lytic phages in the wild [26].

We have shown that although persister *λcI857KnR* lysogens can survive the transient heat induction by being in a dormant state, they cannot survive infection, since the effect of the phage comes into action once persisters switch to normal growth. The difference between the two stresses probably originates from the reversibility of CI857 inactivation under heat induction: bacteria that were dormant during exposure to 42 °C recover an active repressor at the end of the heat treatment [27,28] and therefore survive prophage heat induction. This view is supported by the increase in persistence to prophage induction with UV by photoreactivation, which reverses the UV damage. In contrast, the infection process in phages that do not form lysogens is irreversible: phages might be able to inject their genome into persister bacteria and activate the lytic pathway as soon as bacteria exit from dormancy. Thus, persisters enable differentiating between reversible and irreversible stresses.

### The Effect of Persistence on Predator–Prey Population Dynamics: Predictions from Mathematical Analysis

**Figure 4:**
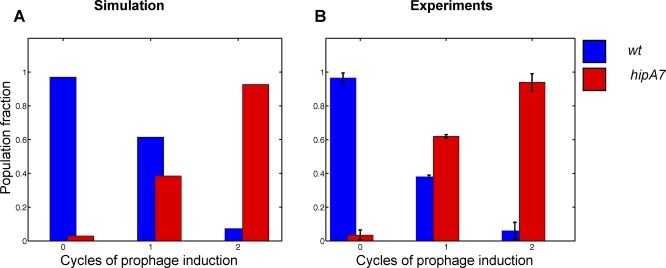
Competition Experiments between Wild-Type and High-Persistence Strains: Simulation and Experiments (A) Simulation of a competition experiment under cycles of prophage heat induction, using the experimental values of the wt and high-persistence *hipA7* survival (Figure 1A). (B) Experiments: The wt *λcI857KnR* and the *hipA7 λcI857KnR* lysogens were mixed in a ratio of 20:1 and exposed to cycles of prophage-inducing conditions. Each prophage induction cycle was followed by O/N growth in culture. Experiments were repeated three times. Error bars represent the standard deviation between three separate experiments.

Our observation that persister cells can survive prophage induction implies that persistence should have a clear effect on the population dynamics of bacteria and phages. This can be shown in a model of the dynamics of two competing populations under cyclic exposure to prophage induction: a bacterial population with higher persistence should quickly overcome a low-persistence strain (Figure 4A). In order to test this prediction, we performed a competition experiment in which a high-persistence strain (*hipA7*) and a low-persistence strain (wt), both carrying the *λcI857KnR* prophage, were mixed and exposed to cycles of prophage-inducing conditions. The results of three independent competition experiments are plotted in Figure 4B; as predicted by the model, the high-persistence strain rapidly took over the low-persistence strain, showing the advantage conferred by persistence in prophage-inducing conditions. These observations, together with evidence of increased prophage induction in biofilms [29] and the role of prophages on biofilm development [30], suggest that persistence might have been selected under the pressure of prophage induction to benefit both bacteria and prophages.

Whereas the degree of persistence was seen to dramatically affect population dynamics in prophage induction, it would seem that its effect on lytic infection should be insignificant, as we observed no increase in the survival of the high-persistence population. However, the extremely long delay observed in the lysis of the subpopulation of persister cells may affect the long-term dynamics of the interaction between phages and their hosts. In order to evaluate the effect of this delay, we introduced it in a mathematical model of the bacterial–phage interaction. Modeling of the population dynamics of the host–phage system is usually based on predator–prey models such as the Lotka-Volterra equations. However, the oscillations predicted from the model are not always found experimentally, and many systems end up either in extinction or stability. Several additions to the Lotka-Volterra equations have been studied, among them logistic growth and time delays. One class of models that has been extensively studied considers the stabilizing effect of spatial heterogeneity on the population dynamics [31,32]. In our case, we do not have spatial heterogeneity; however, the switching between persister and normal cells can be mapped on an effective “migration” model between two “patches” with different conditions. Patch 1, in which growth of the prey is fast (normal bacteria), and patch 2, in which growth of the prey is slow (persisters). Preys can travel between the patches with rates that correspond to the switching rates between persister and normal states. Predators (phages) diffuse rapidly between the patches. A thorough analysis of the equilibrium points of the general equations describing similar processes for symmetrical diffusion between patches as well as their stability has been done in [33]. The analysis for nonsymmetrical diffusion, more appropriate for the description of persistence, is beyond the scope of this work and left for future work. We show below simplified dynamics, illustrating the potential effects of Type I persisters on the host–phage interaction. Type I persisters are generated at stationary phase. When diluted in fresh medium, they switch to normal growth on a time scale of 10 h in our system. No additional persisters are formed. The equations describing this system take into account the unidirectional migration from persister to normal states:

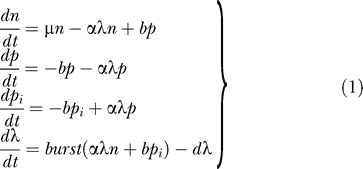
where *n* represents normal bacteria; *p*, persister bacteria; *p_i_*, infected persister bacteria; μ, growth rate of normal bacteria; *b*, switching rate from *p* to *n*; *α*, rate of phages attachment; *λ*, phages; *burst*, burst size, and *d*, dilution/death rate of phages

**Figure 5:**
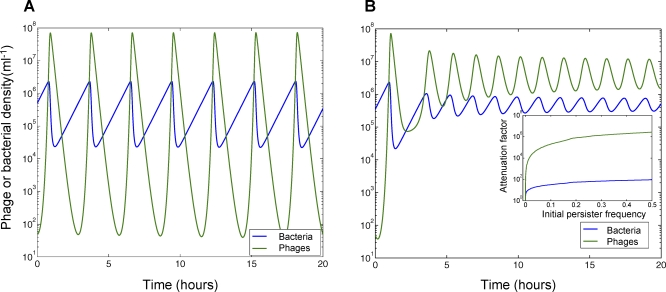
Effect of Persistence on the Dynamics in a Lotka-Volterra Predator–Prey Model (A) Population size without persisters; green: phages; blue: bacteria. (B) Same as (A) but in a population with persisters: the population oscillations are significantly reduced. Inset: reduction in population oscillations as function of the persisters fraction.

Persisters (*p*) are as susceptible to infection as normal bacteria (*n*). Infected persisters (*p_i_*) release phages only upon switching to the normally growing state, at a rate constant *b*, as measured in our experiments. Here, we model a phage infection starting from a small number of phages, typically of the order of the burst size, as expected from a typical infection. Free phages are rapidly diluted out. Simulations of infected populations without and with persisters are shown in Figure 5A and 5B, respectively. For similar initial conditions, the bacterial population undergoes very large oscillations in the absence of persisters, whereas these oscillations are partially reduced by the slow release of phages by persisters. Type I persisters generated at stationary phase alter the dynamics by continuously releasing phages on a slow time scale, thus preventing sustained large oscillations. For wt E. coli, a Type I persister fraction of nearly 1% has been measured [11]. This fraction was sufficient to reduce the amplitude of the oscillations by nearly two orders of magnitude in our simulations. This indicates that population dynamics of wt populations can be affected by the host heterogeneity and that this heterogeneity should be taken into account in complete models of the predator–prey interaction.

### Conclusion

We have shown that the nongenetic individuality in the exit from stationary phase found in populations that persist intensive antibiotic treatments can dramatically affect the interaction between bacteria and λ-phages. Dormancy protects persister cells from prophage induction. Thus, persistence to prophage induction could prevent the eradication of lysogenic bacterial populations by prophage induction, and benefit both bacterial hosts and phages in conditions that trigger prophage induction, such as in biofilms and in stressful environments. However, we have shown that lytic phages are able to infect persistent cells, wait for their switching back to the normally growing state, and then eliminate them by lysis. This implies that bacteria persistent to antibiotic treatments might be targeted by phages [34]. Furthermore, we show that the heterogeneity of the host can significantly alter the predator–prey population dynamics and might be relevant in various other systems. Our results suggest that the ability of phages to infect bacteria under various physiological conditions enables them to benefit from the host heterogeneity under both lysogenic and lytic conditions. Further studies on the influence of nongenetic individuality on the host–phage interaction might shed light on the coevolution of this system.

## Materials and Methods

### Media and chemicals.

LB was used as growth medium for all experiments. Serial dilutions were performed in this medium for bacterial plating. Ampicillin (Sigma) was used at 100 μg/ml; tetracycline (tet) at 12.5 μg/ml; and kanamycin at 30 μg/ml. The penicillinase (Sigma) stock was suspended at 5,000 U/ml in sterile water and kept at 4 °C. The stock solution was diluted 1:40 in water and filter sterilized before use.

### Bacteria, phages, and plasmids.

The plasmid carrying *pR′-tR′-gfp* fusion was described in the work of Kobiler et al. [20]. Briefly, a plasmid with pBR322 origin of replication and ampicillin resistance was used; the *pR′-tR*′ region from the λ genome was amplified by PCR using primers 5′-CGCGGATCCGATGCAAAGGCGTCGGCTATTC and 5′-GCGTCTAGACGCCGACTCTTCACGATTATC, and then inserted upstream of the GFP coding sequence (CDS) on the plasmid. Bacteria and phages strains are summarized in Text S1.

### Growth conditions.

Single colonies were diluted into fresh LB with the appropriate antibiotics. For infection experiments, 0.2% maltose was added. Cells were grown overnight (O/N) at 32 °C, with shaking. Cultures were diluted 1:50 into fresh LB with the appropriate antibiotics and grown for 1 h. To keep growth conditions constant between the different experiments, cultures were transferred to a 42 °C bath for 30 min (unless indicated otherwise), with shaking.

### Heat-induction assay.

MG1655*(λcI857KnR)* and MG1655A7*(λcI857KnR)* lysogens were incubated at 42 °C for the required time. At the indicated time points, samples were taken and plated at serial dilutions on LB-kanamycin (LB-Kan) plates. Experiments were repeated at least three times.

### Competition experiments.

The MG1655A7 high-persistence strain and the wt strain MG1655 both carrying the *λcI857KnR* lysogen were mixed in a ratio of 1:20 and exposed to cycles of prophage-inducing conditions. The total number of surviving bacteria was determined by plating and CFU counting on LB-Kan plates. The number of MG1655A7 bacteria in the population was determined by plating and CFU counting on LB-Kan+tet plates, before and after each heat cycle. Each prophage induction cycle was followed by growth O/N in culture and freezing at −80 °C in 15% glycerol. In order to test the neutrality of the tetracycline resistance marker, a MG1655 *λcI857KnR* lysogenic strain with the same tetracycline resistance marker was constructed (MG21 *λcI857KnR*). The competition experiment in the same conditions showed the neutrality of the marker.

### Infection.

MG1655*/pR′-tR′-GFP* and MG1655A7*/pR′-tR′-GFP* bacteria were grown as described above. Cells were concentrated twice by centrifugation at 5,000 *g* for 5 min and resuspended in fresh LB to an approximate concentration of 10^10^ cells/ml. A total of 10^8^ cells were transferred into an Eppendorf tube, and 10^9^ phages (or the same volume of LB for the control) was added. After 30-min incubation on ice for adsorption, the infected cells were transferred to 37 °C for 5 min to facilitate DNA injection. Serial dilution plating was carried out and CFU determined. Alternatively, two rounds of centrifugation at 2000 *g* for 10 min and washing were done before plating.

### Microscopy.

*Observation chambers,* A polydimethylsiloxane (PDMS) square mold was cut out of cured Sylgard 184 (Dow Corning) layer (thickness: approx. 2 mm). The mold was filled with melted LB-agarose with appropriate antibiotics. Bacteria (1 μl) were put on a coverslip (#1.5) and covered with the solidified LB-agarose inside the PDMS mold. The whole chamber was sealed with a thin layer of PDMS to avoid dehydration of the LB-agarose, without blocking oxygenation.

*Time-lapse microscopy.* The PDMS chambers were monitored using a Leica DMIRBE inverted microscope system with incubator box (LIS), automated stage, and shutters. Autofocus and image acquisition was done using custom macros in ImagePro/Scope-Pro (Media Cybernetics) to control the microscope, stage, shutters, and camera. Multiple different locations were monitored in parallel for phase contrast and fluorescence on the same chamber. Images were acquired using a 40×/1.5 or 63× long-range air objective and a cooled charge-coupled device (CCD) camera (Orca; Hamamatsu) and processed with ImagePro and ImageJ (http://rsb.info.nih.gov/ij/). Fluorescence images were acquired with minimal excitation to minimize bleaching and photodamage. This was checked in situ by exposing one of the multiple locations as control to only 1/10 of the integrated illumination dose. No significant difference was noticed at the control location.

Unless otherwise indicated, microscopy was carried out at 32 °C.

Transient heat induction under the microscope was done on a custom-made heated stage insert warmed to 42 °C.

*Single cells microscopy experiments analysis.* Analysis of the videos was done with ImageJ, by tracking cells in sequential frames and measuring their mean fluorescence.

### Numerical Simulations.

Simulations of Lotka-Volterra equations with and without persisters were performed using Matlab software (The MathWorks).

## Supporting Information

Figure S1 Persistence to Prophage Induction Depends on the Inoculum Size Survival percentage to prophage heat induction for wt and *hipA7 λcI857KnR* lysogens. Cultures were started with different inocula and exposed to heat at the same optical density (OD).
(14 KB PDF)

Figure S2 Persistence to Prophage Induction by UV Survival percentage following UV irradiation of lysogenic *λc+KnR* strains. The high-persistence lysogens have a marked higher survival to prophage induction when compared to the wt strain.
(10 KB PDF)

Text S1 Additional Experiments and Methods (1 KB DOC)

Text S2 Time Stamps of Videos S1 and S2
(1 KB DOC)

Text S3 Time Stamps of Video S3
(1 KB DOC)

Video S1 Single-Cell Observations of Persistence Following Heat Induction for the Wild-Type *λcI857KnR* Lysogens Superposition of phase-contrast and fluorescence images acquired by time-lapse microscopy of wt strain carrying the *λcI857KnR* prophage during and after heat induction. Fluorescence expression of the *pR′-tR′-gfp* is monitored. At *t* = 0, lysogenic bacteria are exposed to 42 °C for 80 min. The temperature is then set back to 32 °C. Videos were acquired in parallel at several different locations. At the end of the measurement, no survivors are observed in the wt. The times between frames are indicated in Text S2. Images were flattened and filtered using High Gauss (ImagePro).
(803 KB AVI)

Video S2 Single-Cell Observations of Persistence Following Heat Induction for the *hipA7 λcI857KnR* Lysogens Superposition of phase-contrast and fluorescence images acquired by time-lapse microscopy of the *hipA7* strain carrying the *λcI857KnR* prophage during and after heat induction. Fluorescence expression of the *pR′-tR′-gfp* is monitored. At *t* = 0, lysogenic bacteria are exposed to 42 °C for 80 min. The temperature is then set back to 32 °C. Videos were acquired in parallel at several different locations. At the end of the measurement, no survivors are observed in the wt (Video S1), whereas a number of dividing cells can be seen in the *hipA7* strain. The times between frames are indicated in Text S2. Images were flattened and filtered using High Gauss (ImagePro).
(803 KB AVI)

Video S3 Exit from Dormancy Is Required for Phage-Mediated Lysis Superposition of phase-contrast and fluorescence images acquired by time-lapse microscopy of the *hipA7 λcI857KnR* lysogens after heat induction, with *λimm434cI3003* phage superinfection. Fluorescence expression of the *pR′-tR′-gfp* is monitored. At the end of the measurements, no survival is observed in the superinfected cells, and late onset of fluorescence is seen. The times between frames are indicated in Text S3. Images were flattened and filtered using High Gauss (ImagePro).
(1.74 MB AVI)

## Abbreviations

CFU: colony forming unit
GFP: green fluorescent protein
wt: wild-type
