# Supplementary material for: Nongenetic Individuality in the Host–Phage Interaction
Source: PLoS Biol. 2008 May 20;6(5):e120. doi: 10.1371/journal.pbio.0060120 (PMC2386839; doi:10.1371/journal.pbio.0060120)
Supplement: Supplementary file 1 [file pbio.0060120.sd001.pdf]

Supporting information

Persistence to prophage heat induction is a transient phenotype

We exposed *λcI857KnR* lysogenic bacteria to several cycles of heat induction and saw no increase in the persistence fraction. *λcI857KnR* lysogenic bacteria were grown O/N from a single colony in 10 ml LB-kanamycin (30 μg/ml) at 32°C with shaking. Cultures were diluted 1:50 into fresh LB-kanamycin and grown for another 30 min at 32°C. Heat induction of the prophage was done by exposure to 42°C in a bath for 30 min. Samples were taken before and after exposure to 42°C and plated to determine CFU. After the heat induction, bacteria were diluted 10^4 into fresh LB-kanamycin and grown O/N at 32°C with shaking for the next cycle.

%survivals	2nd cycle	3rd cycle
wt(λcI857KnR)	0.36	0.1
hipA7(λcI857KnR)	34	33

Table S1: Survival to heat induction for *wt* and *hipA7* lysogenic bacteria exposed to several cycles of heat induction.
No increase in survival was observed after 3 cycles.

The results show no increase in the survival during cycles of exposure to heat induction (Table S1). Therefore, persistence to prophage heat induction is not genetically acquired nor passed on to the next generations.

Dependence of persistence to prophage heat induction on the inoculum's size

Type I persisters are generated at stationary phase. Once the culture is diluted into fresh medium and grows exponentially, no new Type I persisters are generated. Therefore, the number of persisters depends on the size of the population inoculated from the initial stationary phase culture and not on the total number of bacteria in the culture.

In order to test whether the persistence to prophage heat induction showed the same feature, we measured the survival for the *wt* and *hipA7* lysogenic strains for two different inoculum sizes. *λcI857KnR* lysogenic bacteria were grown O/N in LB-kanamycin at 32°C with shaking. The culture was diluted 1:50 into fresh LB-kanamycin, resulting in an inoculum of 10^7 bacteria. The culture was grown at 32°C

for another hour before the prophage heat induction. A culture with an inoculum of 10^3 bacteria was obtained by diluting the same O/N culture 1:10^6, and grown till the OD reached 0.01-0.02. All cultures were exposed to prophage induction at the same OD, in a 42°C bath for 80 min., with shaking. Serial dilutions were plated on LB-kanamycin plates before and after exposure to 42°C.

The results are summarized in Fig. S1. For both *wt* and *hipA7* lysogens, there is a marked decrease of persistence with inoculum's size, as expected from Type I persistence.

Absence of persistence to lytic phage infection is not due to infection after plating

As long as the stressful conditions are maintained, persistence will not result in increased survival. For example, persistence to ampicillin is observed only when the antibiotic is washed away and the number of survivors determined by plating. Therefore, in order to measure persistence to phage infection, we need to ensure that we stop the exposure to phages after plating. We tested whether the absence of survival to lytic phage infection was due to the initial adsorption of phages, or to infection after plating. For this purpose, we used a sensitive population of control bacteria that did not undergo the adsorption step: *hipA7* cells were grown and infected with *λcI60* as explained in Material and Methods. A constant number of non-infected sensitive control bacteria were added to different dilutions of the infected *hipA7* cells. These mixtures were plated on LB plates and incubated O/N at 32°C. The sensitive control bacteria (MGY) carried the YFP gene fused to a constitutive promoter. Therefore, control colonies could be discriminated from *hipA7* colonies by fluorescence. The total number of CFU of the control bacteria stayed within the experimental error for dilutions above 10^{-4} (Table S2). Thus, for this range of dilutions, no significant infection occurred after plating. Therefore, plating results were extracted from dilutions at least as high as 10^{-4}, consistent with [5].

We also tested whether secondary infections took place in our microscopy experiments and observed no secondary infections. This was achieved by repeating the control experiment described above under the microscope. The uninfected control bacteria marked by fluorescence did not undergo secondary infections.

Dilution	CFU/plate
10^{-2}	57±12
10^{-3}	73±14
10^{-4}	124±19
10^{-5}	129±5
No Φ	154±10

Table S2: **Effect of dilution on plating efficiency of sensitive bacteria.** A constant number of sensitive control bacteria were mixed with different dilutions of infected bacteria and plated in triplicates. No significant decrease in plating efficiency was observed up to 10^{-4} dilution. CFU were determined for the control bacteria only. The table shows the results from triplicates.

Prophage induction of *λc+KnR* by UV

In order to test whether persistence affects also the outcome of the prophage induction through inactivation of the *wt* cI (*λc+)* by the SOS response, we subjected *wt* and *hipA7* lysogens of *λc+KnR* strains to UV irradiation. Aliquots of O/N cultures were diluted at appropriate dilutions and plated on LB kanamycin plates. After 2 hours of incubation in the dark at 32°C, plates were exposed to a dose of 100 J/m^2 at 258 nm. Plates were incubated in the dark at 32°C. After 4 hours, plates were exposed to visible light under a PL lamp (at 50 cm from four lamps of 9W each) for photoreactivation [1,2] and incubated for 48 hours. Survival was determined by CFU counting. A clear advantage in survival was observed for the *hipA7* lysogenic strain (Fig. S2). In order to test that this advantage was due to the activation of the prophage and not to the direct effect of survival to UV damage, the experiments were repeated for the *wt* and *hipA7* strains without the prophage. No significant difference in survival was measured, showing that in our conditions, the large advantage in survival of the *hipA7* strains is due to persistence to UV prophage induction.

Lotka-Volterra simulation parameters:

b=0.1 h^{-1}: switching rate from p to n

α: 4 $10^{-7}h^{-1}$/bacterium/phage: rate of phages attachment

Burst=50: burst size

d=10 h^{-1}: dilution/death rate of phages

Table S3: Bacteria and Phage strains

Strain	Relevant characteristics	Reference
HM22		[3]
MG1655	*wt* E.coli K-12	*E.Coli* Genetic Stock Center (CGSC#7740)
MG1655A7	MG1655 with *hipA7*; P1 of HM22 in MG1655 using Tet selection	This work
MG21	P1 of HM21 in MG1655 using Tet selection	This work
MGY	MG1655 with pR-YFP fusion and Cam resistance	Balaban NQ *et al* 2004.
λcI857KnR	lambda phage with t.s. repressor, which carries the *ind*$^-$ mutation. and Kan resistance	AB Oppenheim collection
λc$^+$*KnR*	lambda phage with Kan resistance	AB Oppenheim collection
λcI60	Lambda phage with a non active repressor	AB Oppenheim collection
λimm434cI3003	Lambda phage in which the immunity region is replaced with immunity region from phage 434. This phage carries a non	[4]

	active repressor	
MG1655/*pR'-tR'-GFP*	MG1655 carrying the *pR'-tR'*-GFP plasmid	This work
MG1655A7/*pR'-tR'-GFP*	MG1655A7 carrying the *pR'-tR'*-GFP plasmid	This work
MG1655*(λcI857KnR)*	MG1655 lysogenic for *λcI857KnR*	This work
MG1655A7*(λcI857KnR)*	MG1655A7 lysogenic for *λcI857KnR*	This work
MG1655*(λcI857KnR)/pR'tR'*	MG1655 lysogenic for *λcI857KnR*, carrying *pR'tR'*-GFP	This work
MG1655A7*(λcI857KnR)/pR'tR'*	MG1655A7 lysogenic for *λcI857KnR*, carrying *pR'tR'*-GFP	This work
MG1655*(λc^+KnR) /pR'tR'*	MG1655 lysogenic for lambda phage with Kanamycin resistance, carrying *pR'tR'*-GFP	This work
MG1655A7*(λc^+KnR)/pR'tR'*	MG1655A7 lysogenic for lambda phage with Kanamycin resistance, carrying *pR'tR'*-GFP	This work

References

1. Jagger J (1958) Photoreactivation. Bacteriol Rev 22: 99-142.
2. Jacob F (1964) Les Bacteries Lysogenes et la Notion de Provirus. Monographies de l'Institut Pasteur (Masson et Cie, ed).
3. Moyed HS, Bertrand KP (1983) hipA, a newly recognized gene of Escherichia coli K-12 that affects frequency of persistence after inhibition of murein synthesis. J Bacteriol 155: 768-775.
4. Oppenheim AB, Salomon D (1972) Studies on partially virulent mutants of lambda bacteriophage. II. The mechanism of overcoming repression. Mol Gen Genet 115: 101-114.

5. E. Kutter and A. Sulakvelidze (ed.) (2005) Bacteriophages-Biology and Applications ed CRC Press: p. 481.
