# Supplementary material for: Nongenetic Individuality in the Host–Phage Interaction
Source: PLoS Biol. 2008 May 20;6(5):e120. doi: 10.1371/journal.pbio.0060120 (PMC2386839; doi:10.1371/journal.pbio.0060120)
Supplement: Supplementary file 2 [file pbio.0060120.sg001.pdf]

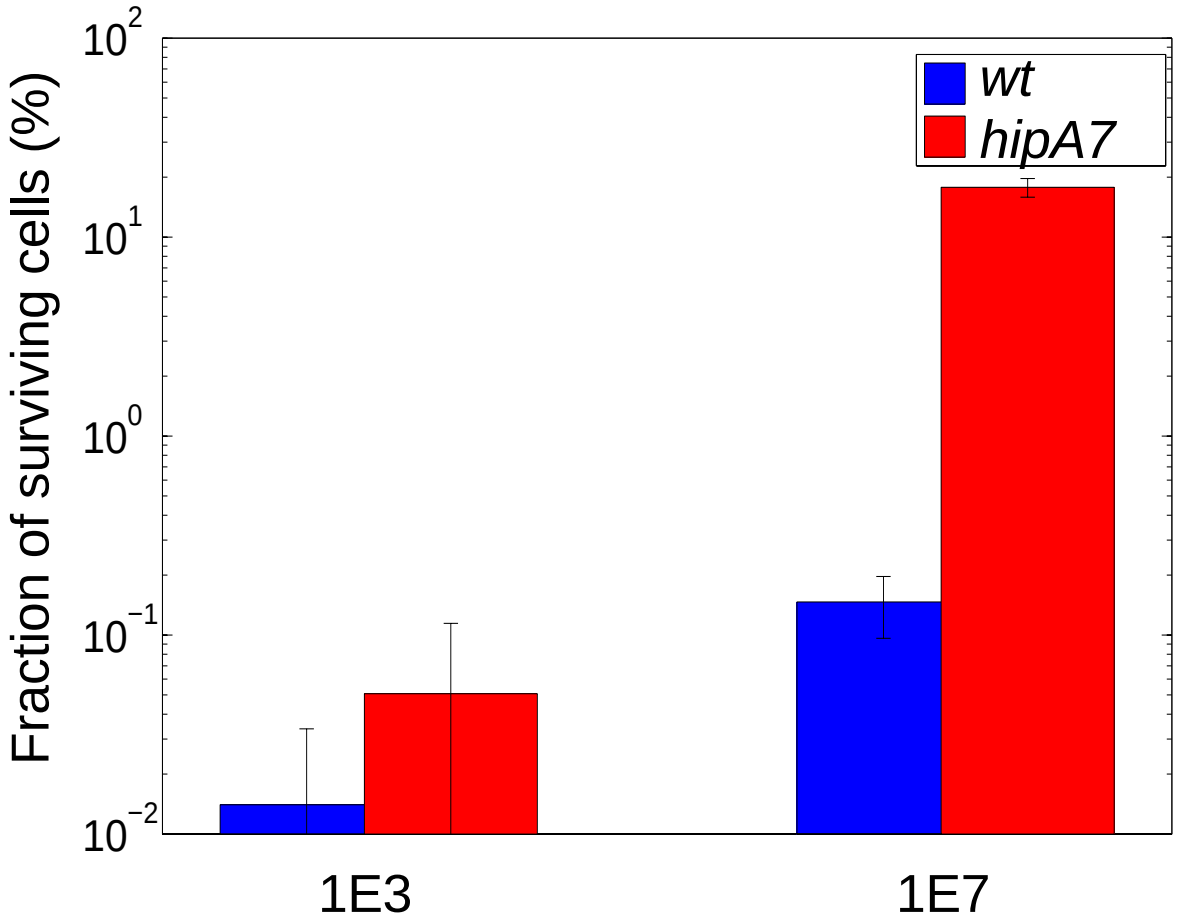

Fraction of surviving cells (%)
10^2
10^1
10^0
10^{-1}
10^{-2}
wt
hipA7
1E3
1E7
