# Supplementary material for: Nongenetic Individuality in the Host–Phage Interaction
Source: PLoS Biol. 2008 May 20;6(5):e120. doi: 10.1371/journal.pbio.0060120 (PMC2386839; doi:10.1371/journal.pbio.0060120)
Supplement: Supplementary file 3 [file pbio.0060120.sg002.pdf]

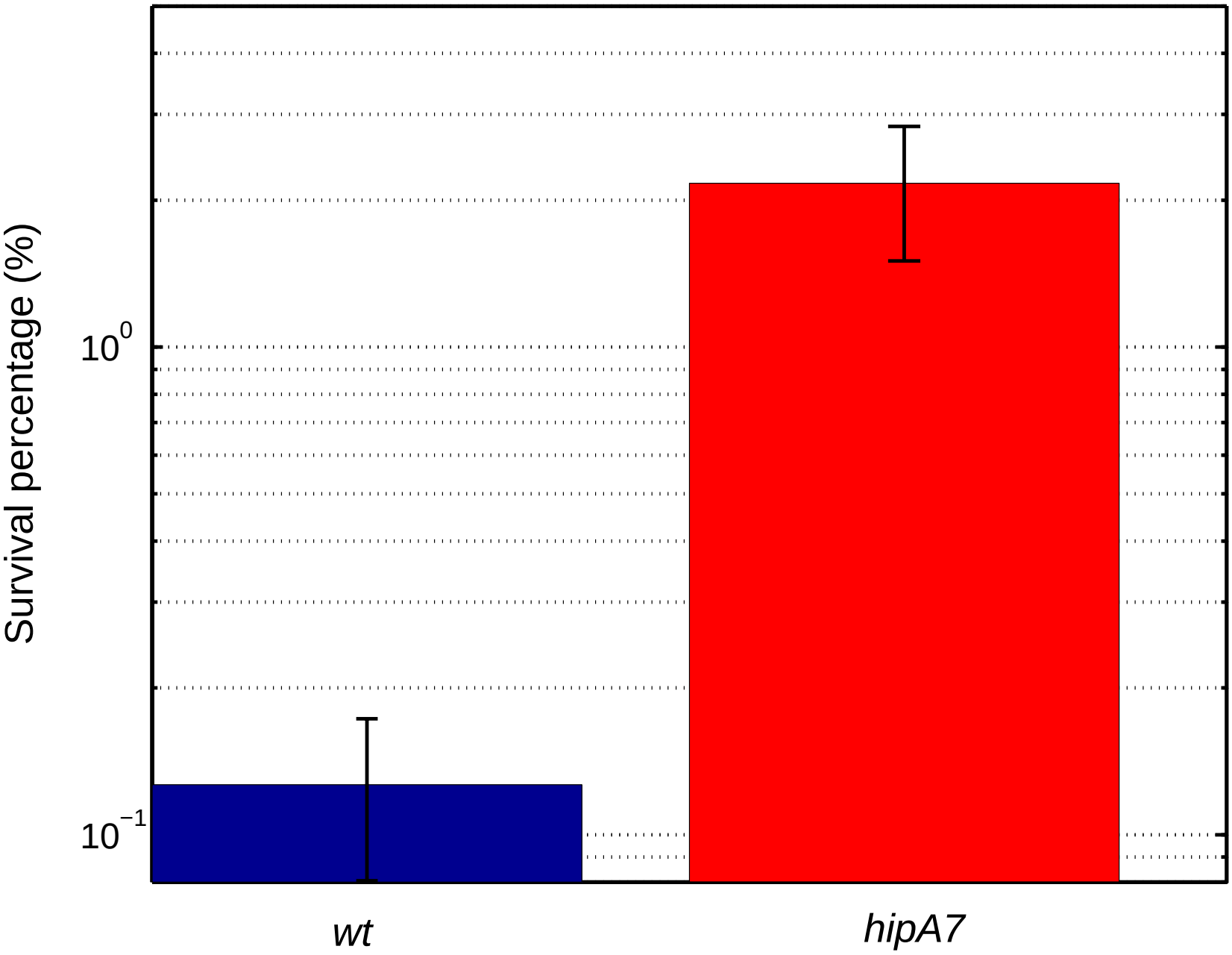

Survival percentage (%)
10^0
10^−1
wt
hipA7
