# Supplementary material for: Nongenetic Individuality in the Host–Phage Interaction
Source: PLoS Biol. 2008 May 20;6(5):e120. doi: 10.1371/journal.pbio.0060120 (PMC2386839; doi:10.1371/journal.pbio.0060120)
Supplement: Supplementary file 4 [file pbio.0060120.sd002.doc]

hour	min

0	0

0	16

0	33

0	49

1	6

1	24

1	57

2	13

3	3

3	19

3	35

3	52

4	8

4	24

4	40

4	56

5	12

5	42

5	44

6	0

6	16

6	32

6	49
