# Supplementary material for: Nongenetic Individuality in the Host–Phage Interaction
Source: PLoS Biol. 2008 May 20;6(5):e120. doi: 10.1371/journal.pbio.0060120 (PMC2386839; doi:10.1371/journal.pbio.0060120)
Supplement: Supplementary file 5 [file pbio.0060120.sd003.doc]

hour	min

0	0

0	13

0	25

0	37

0	50

1	2

1	14

1	26

1	38

1	50

2	2

2	14

2	27

2	39

2	51

3	3

3	15

3	27

3	51

4	4

4	16

4	28

4	40

4	52

5	4

5	16

5	28

5	40

6	29

6	41

6	53

7	5

7	17

7	29

7	41

7	53

11	31

11	43

11	55

12	7

12	20

12	32

20	59

21	11

21	23

21	35

21	48

25	49
